# Role of Interfacial Bonding in Tribochemical Wear

**DOI:** 10.3389/fchem.2022.852371

**Published:** 2022-04-06

**Authors:** Chunsheng Luo, Yilong Jiang, Yangqin Liu, Yang Wang, Junhui Sun, Linmao Qian, Lei Chen

**Affiliations:** ^1^ Tribology Research Institute, State Key Laboratory of Traction Power, School of Mechanical Engineering, Southwest Jiaotong University, Chengdu, China; ^2^ State Key Laboratory of Solid Lubrication, Lanzhou Institute of Chemical Physics, Chinese Academy of Sciences, Lanzhou, China; ^3^ Technology and Equipment of Rail Transit Operation and Maintenance Key Laboratory of Sichuan Province, Southwest Jiaotong University, Chengdu, China

**Keywords:** tribochemical wear, atomic-scale, interfacial bonding, silicon, diamond-like carbon

## Abstract

Tribochemical wear of contact materials is an important issue in science and engineering. Understanding the mechanisms of tribochemical wear at an atomic scale is favorable to avoid device failure, improve the durability of materials, and even achieve ultra-precision manufacturing. Hence, this article reviews some of the latest developments of tribochemical wear of typical materials at micro/nano-scale that are commonly used as solid lubricants, tribo-elements, or structural materials of the micro-electromechanical devices, focusing on their universal mechanisms based on the studies from experiments and numerical simulations. Particular focus is given to the fact that the friction-induced formation of interfacial bonding plays a critical role in the wear of frictional systems at the atomic scale.

## Introduction

Wear of the moving systems often results in material loss, device failure, and excessive consumption of lubricants, which will, thus, result in energy waste and environmental pollution. It has been reported that 5%–7% of the gross domestic product (GDP) is resulted from the wear and relative negative impacts of the moving parts. Hence, it is urgent to understand the possible general mechanism of wear processes and control it for economic benefits and technological advancements. In recent years, high-density memory, advanced cutting tool, precision bearing, micro-electromechanical system (MEMS), optical lens, and integrated circuit (IC) are rapidly developed to stimulate the progress of modern science and technology (Kobatake et al., 2005; Fonseca and Sequera, 2011; Kim et al., 2016; Lincong Liu et al., 2019; Seo, 2021). The micro-/nano-scale wear of materials such as two-dimensional (2D) materials, diamond and DLC films, silicon and silicon oxides, and metals has thus attracted more and more attention, especially the wear processes between the contact surfaces that are usually sensitive to the work environment of the devices, for example, humidity, atmosphere, liquid, and lubricant (Kato and Adachi, 2002; Wang et al., 2009; Kim et al., 2012; Kumar et al., 2018; Zhe Chen et al., 2018).

Despite the typical two-dimensional (2D) materials, graphene and hexagonal boron nitride (h-BN) significantly enhance the wear-resistance of the contact surfaces in humidity (Lee et al., 2010; Cao et al., 2014; Wang and Duan, 2018), while molybdenum disulfide (MoS_2_) materials are often aggravated (Picas et al., 2006; Li et al., 2019). As the hardest coating materials, the diamond and diamond-like carbon (DLC) films suffer from remarkable wear due to the tribochemical reactions under high temperature and in vacuum but wear-free in inert gas environment (Erdemir and Martin, 2018; Rajak et al., 2021). The wear behaviors of silicon, silicon oxide, and silicate surfaces also depend on tribochemical reactions, which largely dominate the failure of MEMS and the manufacturing precision of IC (Katsuki, 2009; Bingjun Yu et al., 2012; Wang et al., 2013; Lei Chen et al., 2017a) and the dimensional accuracy of the optical lens (Lin Wang et al., 2021). For ceramics (e.g., SiC and Si_3_N_4_), the wear is suppressed in humidity, water, and ionic liquid due to the formation of tribo-layers induced by the tribochemical reactions between the sliding interfaces (Khanna et al., 2017; Qin et al., 2018; Chen et al., 2019; Ge et al., 2019; De Fine et al., 2021). Moreover, the wear behaviors between the contact surfaces of the metals or alloys in humidity or with lubricants are suppressed when the oxide layers are arisen by the tribochemical reactions (Cai et al., 2009; Barthel et al., 2012; Fukuda et al., 2019; He et al., 2021). Accordingly, the previous experiments have evidenced that tribochemical reactions should play critical roles in the micro-/nano-scale wear (or material removal) of friction systems. However, little attention is paid to the detailed processes and mechanisms of tribochemical wear at an atomic scale.

Fortunately, many methods of theoretical simulations have been developed to understand the vivid wear processes in the friction systems at the macro/nano scale (Zhang and Mylvaganam, 2006; Popov and Psakhie, 2007; Cui and Zhang, 2017; Martini et al., 2020). One method of movable cellular automata (MCA), due to the material model incorporating more details of material behavior, is usually used to study the wear and fracture behaviors by the Psakhie group (Psakhie et al., 2013; Dimaki et al., 2020), but MCA is inadequate to study the tribochemical wear behaviors of the interfacial bonding at an atomic scale. Molecular dynamics (MD) simulation as a theoretical investigation method has the advantage of providing the interfacial bonding behaviors while resolving all the positions, velocities, forces, and bonding states of the atoms (Zhang et al., 2006). Wang et al. (2017a) revealed that this interfacial C–C bond may induce the dissociation of C–C bonds in DLC films during friction as an initial step of structure failure. Lei Chen et al. (2018) demonstrated the detailed interfacial bonding for the tribochemical wear of monocrystalline silicon at the atomic scale. Hence, based on the experiments and MD simulations, a clear grasp of the wear mechanism would be desired to predict and control the wear behaviors of the friction systems at all length scales from nanoscale to macroscale.

The present article provides a brief review of the micro-/nano-scale wear behaviors of typical materials, focusing on their underlying wear mechanisms and processes based on the results of the experiments and numerical simulations. The materials concerned here include 2D materials (graphene, h-BN, and MoS_2_), carbon bulk materials (diamond and DLC films), silicon-based materials (silicon and silicon oxide, silicon-based ceramics, and silicate glasses), and metals (Al and Cu). It should be noted that the tribochemical wear closely depends on ambient medium and chemical properties of the substrate surface and counterface. However, here, we only focus on the recent advancements of the micro-/nano-scale wear related to the ambient medium.

## Two-Dimensional Materials

**FIGURE 1:**
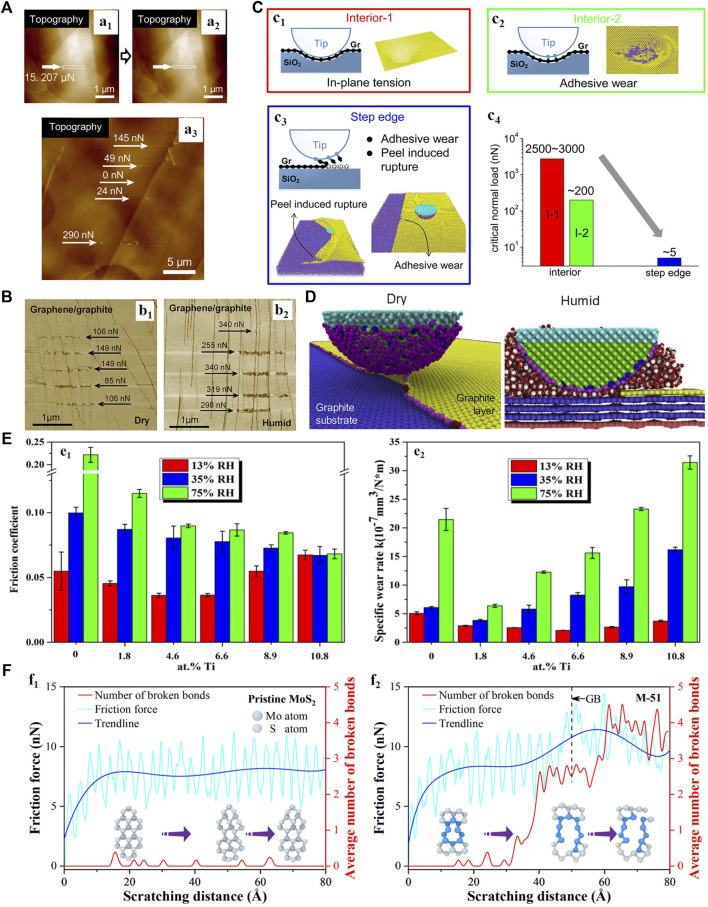
**(A)** Comparative scratch tests of graphite in the interior region and at the step edge of the graphene monolayer. No remarkable changes in the topographies before (a_1_) and after (a_2_) scratching at 15.207 μN indicates no wear occurring in the interior region. Differently, the wear of the step edge initiates at a normal load of 24 nN (a_3_). Adapted from Qi et al. (2017) **(B)** Scratch tests at the step edges of the monoatomic graphene layer on a graphite surface in dry (b_1_) and humid (b_2_) conditions. Adapted from (Qi et al., 2018) **(C)** MD simulations of the scratching tests showing the wear of in-plane graphene with tension damage (c_1_) or with abrasive wear (c_2_) initiation at much larger critical loads (c_4_) compared to the fracture of the step edge (c_3_). Adapted from Qi et al. (2017) **(D)** MD simulations of scratching a diamond tip across the step edge of the monoatomic graphene layer on a graphite substrate. Adapted from Qi et al. (2018) **(E)** Friction coefficient and wear rate of MoS_2_ and MoS_2_/Ti composite coatings in different humidity. Adapted from (Li H. et al., 2017) **(F)** MD simulations about the change of friction force and the average number of broken Mo‐S bonds during the scratching process of the MoS_2_ without (f_1_) and with (f_2_) defects. Adapted from Wei et al. (2021).

Due to their atomic thickness and ultralow shear strength, the typical 2D materials such as graphene, h-BN, and MoS_2_ are often used as solid-lubricant coating for the nanoscale devices to suppress the wear (Berman et al., 2014; Spear et al., 2015; Berman et al., 2018; Liu L. et al., 2019). However, 2D coatings often fail to maintain its integrity when they are subjected to macro-scale tribological tests, and the wear behaviors are sensitive to the surrounding environment (Marchetto et al., 2012; Huang et al., 2017; Zheng-yang Li et al., 2017). To better understand the wear behavior depending on the scale and environment, Qi et al. (2017); Qi et al. (2018) performed a series of atomic force microscope (AFM) scratch tests and found a substantially lower wear resistance at the step edge of a monolayer graphene sheet compared to that obtained within the interior region (Figure 1A), but the wear resistance at the step edge is enhanced in humidity (Figure 1B). Assisted with the MD simulations, the authors further explained that the weaker strength at the step edge is attributed to the formation of the C–C covalent bonds between the tip atoms and the nonterminated carbon atoms at the edge of graphene (Figure 1C), while the involving humidity passivates the dangling bonds at the edges or defects to improve the wear resistance of graphene (Figure 1D). Based on the friction tests using the isotopically labeled water, Rietsch et al. (2013) had also found that the sensitive wear behavior in humidity is attributed to the passivation of the dangling bonds by the adsorption of water molecules. Furthermore, the wear due to the synergetic actions of interlocking and pushing between the tip and graphene also occurs at the grain boundaries (GBs) (Zhang et al., 2019) and point defects (Zheng and Duan, 2019). The wear resistance enhancement with the increase of RH was also observed for the h-BN materials, and this phenomenon was mainly attributed to the passivation of the dangling bonds at the edges or defects, which is similar to the tribological mechanism of graphene (Cao et al., 2011; Li and Zeng, 2012).

Unlike graphene and h-BN, MoS_2_ favors the lubrications in water-/oxygen-deficient environments. For instance, Li et al. (2017) found that the wear resistance decreasing at higher RH conditions was found for the MoS_2_ and MoS_2_/Ti composite coatings (Figure 1E). It has been widely accepted that the lubricity loss of MoS_2_ in humid environments is mainly attributed to the chemical oxidation of MoS_2_ layers activated by water vapor, which results in the formation of MoO_3_ with worse frictional profiles (Windom et al., 2011; Curry et al., 2017; Uzoma et al., 2020). Other studies focus on the enhanced molecular interactions of water with the layers as the fundamental mechanism behind the observed loss of MoS_2_ lubricant properties (Levita et al., 2016; Levita and Righi, 2017). Recently, Wei et al. (2021) investigated the effect of GB defects on the tribological properties of MoS_2_ using the MD simulations. They demonstrated that the wear resistance of MoS_2_ with GB defects degenerates owing to the combined effects of shearing and interfacial bonding between the tip and MoS_2_ atoms, as shown in Figure 1F.

## Diamond and DLC Films

**FIGURE 2:**
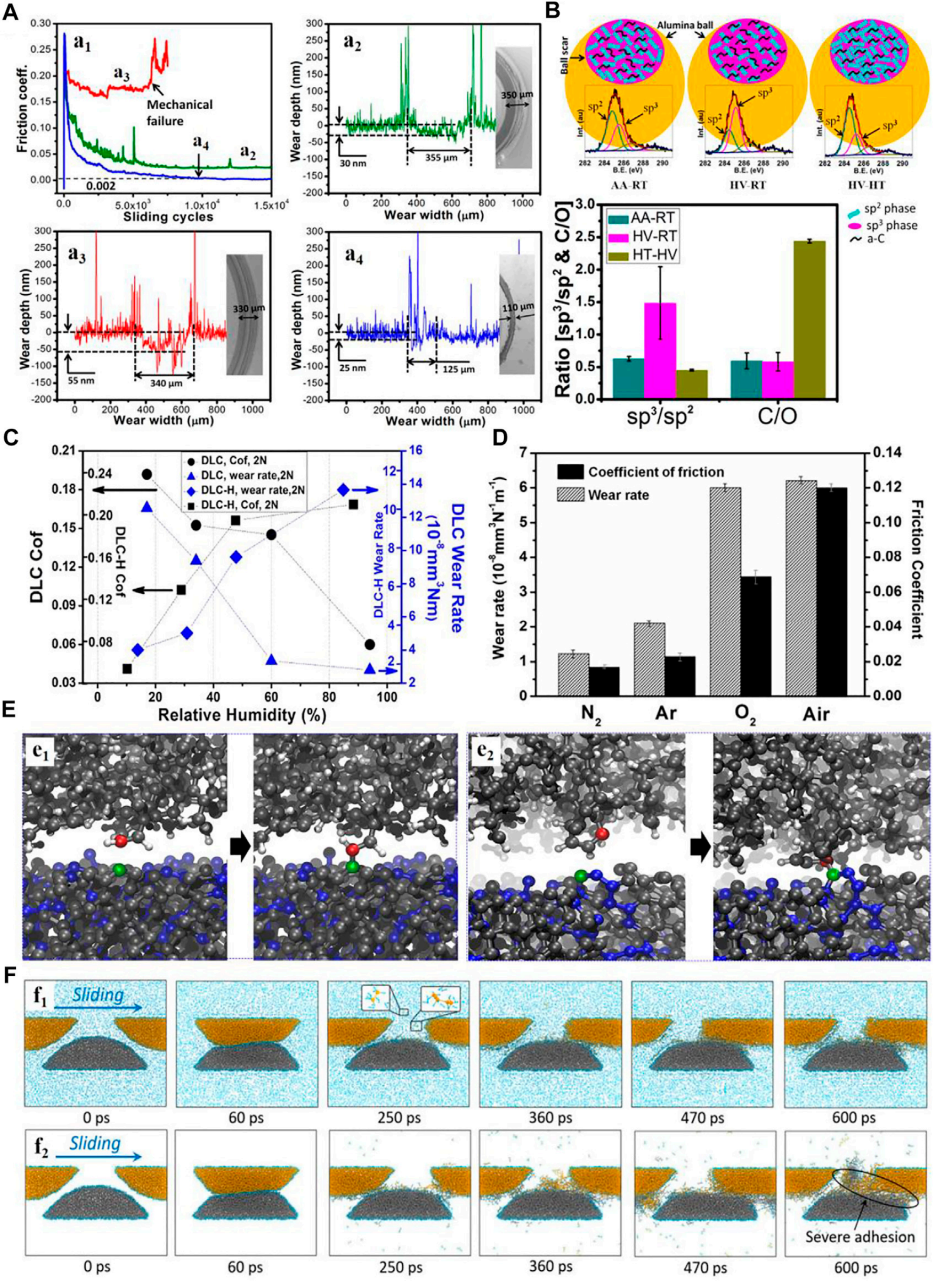
**(A)** Friction behaviors of the UNCD NW film under different experimental conditions (a_1_) and the profiles of the wear track formed in the conditions of AA-RT (a_2_), HV-RT (a_3_), and HV-HT (a_4_). Adapted from Rani et al., 2018b **(B)** Raman spectra measured in the ball scars formed in the different conditions and the calculated ratios of sp^3^/sp^2^ and C/O. Adapted from Rani et al., 2018a **(C)** Steady-state friction coefficients and wear rates of the DLC-H and DLC films depending on the RH. Adapted from Shi et al., 2017a **(D)** Average friction coefficients and wear rates of the DLC-H/Al_2_O_3_ counterparts in N_2_, Ar, O_2,,_ and humid air (RH ∼ 37%). Adapted from Shi et al., 2017b **(E)** Interfacial bonds forming between the carbon atoms at the DLC-H coating probe surfaces and the carbon atoms in a diamond grain (e_1_) or within the grain boundary (e_2_) at the UNCN surface. Adapted from Vahdat et al., 2014 **(F)** Snapshots of the sliding behaviors of hydrogen-free DLC asperities in the (f_1_) hydrogen gas environment and (f_2_) vacuum. Adapted from Wang et al., 2020.

As one of the hardest solid-lubricant materials, diamond or DLC films has been attracting enormous attention in the field of anti-wear design (Kumar et al., 2011; Kumar et al., 2013; Erdemir and Martin, 2018; Tyagi et al., 2019). However, the wear resistance of diamond and DLC films is dependent on humidity and gaseous environment (Manimunda et al., 2017; Huo et al., 2018; Wu et al., 2018; Yunhai Liu et al., 2019; Jingjing Wang et al., 2019; Yu et al., 2020; Latorre et al., 2021; Wang and Komvopoulos, 2021). Notably, the improved wear properties of diamond and DLC films are closely related to the surface passivation or shear-induced graphitization (Bouchet et al., 2015; Rani et al., 2018a; Rani et al., 2018b). As shown in Figure 2A, the tribological behaviors of the ultra-nanocrystalline diamond nano-wall (UNCD NW) films are different in the atmosphere under room temperature (AA-RT), high vacuum under room temperature (HV-RT), and high-temperature (HV-HT). For the conditions of AA-RT and HV-HT, the friction keeps constant and relatively low after the initial running-in processes (a_1_), and the surface wear of the UNCD NW film is extremely weak (a_2_-a_4_). The results of Raman spectra indicate that the slight wear of films is due to passivation of the dangling bonds through the atmospheric water vapor and graphitization of the contact interfaces in AA-RT, and graphitization is the dominating mechanism for the ultrahigh wear resistance of films in HV-HT (Figure 2B) (Rani et al., 2018a). The effect of atmosphere on the wear of DLC films strongly depends on the hydrogen content. Generally, the surface damage of the DLC film without hydrogen is suppressed at high RH due to water lubrication, whereas the wear of the hydrogenated DLC (DLC-H) film is facilitated with an increase of RH (Figure 2C) (Shi et al., 2017a). Similarly, Figure 2D shows that the tribological properties of the DLC-H film become serious by involving active species (oxygen or H_2_O) (Shi et al., 2017b). In those processes, a nano-scale carbonaceous tribo-layer was induced by various tribochemical reactions (such as bond cleavage, migration, and rearrangement of interfacial atoms and bond-formation) in the contact area (Xinchun Chen et al., 2017). We observed the friction-induced nano-crystallites of graphene for the tribo-layer between the DLC-H and steel sliding pairs based on the TEM characterizations (Liu et al., 2021a; Liu et al., 2021b). To better understand the processes of the tribochemical wear at the atomic scale, Vahdat et al. (2014) explored the wear mechanism of DLC-H films based on the MD simulations. They observed the formation of interfacial bonds at under-coordinated atomic sites between the DLC-H coating probes and UNCN samples. Both the cases of the carbon atoms on the surface of a diamond grain (e_1_) and within a grain boundary (e_2_) are consistent with the atom-by-atom removal under the association of interfacial bonding (Figure 2E). We further revealed that this interfacial C–C bond may induce the dissociation of C–C bonds in the DLC films during friction as an initial step of structure failure (Wang et al., 2017a). Furthermore, we demonstrated that the adhesive wear induced by the interfacial C–C bonds was suppressed in the hydrogen gas environment (f_1_) compared to that in vacuum (f_2_), as shown in Figure 2F (Wang et al., 2020). In addition, two different types of tribochemical reactions were revealed at the DLC or DLC-H friction interface: one is the triboemission reaction of hydrocarbon molecules which causes the depletion of surface hydrogen terminations and hence accelerates the interfacial bond formation and resulted in atomic-level adhesive wear, while another one is the dissociative desorption of the environmental gases (i.e., H_2_ molecule) which replenish the depleted hydrogen terminations so that the interfacial bond formation and the interfacial bonding–induced atomic-level wear are suppressed.

## Silicon and Silicon Oxide

**FIGURE 3:**
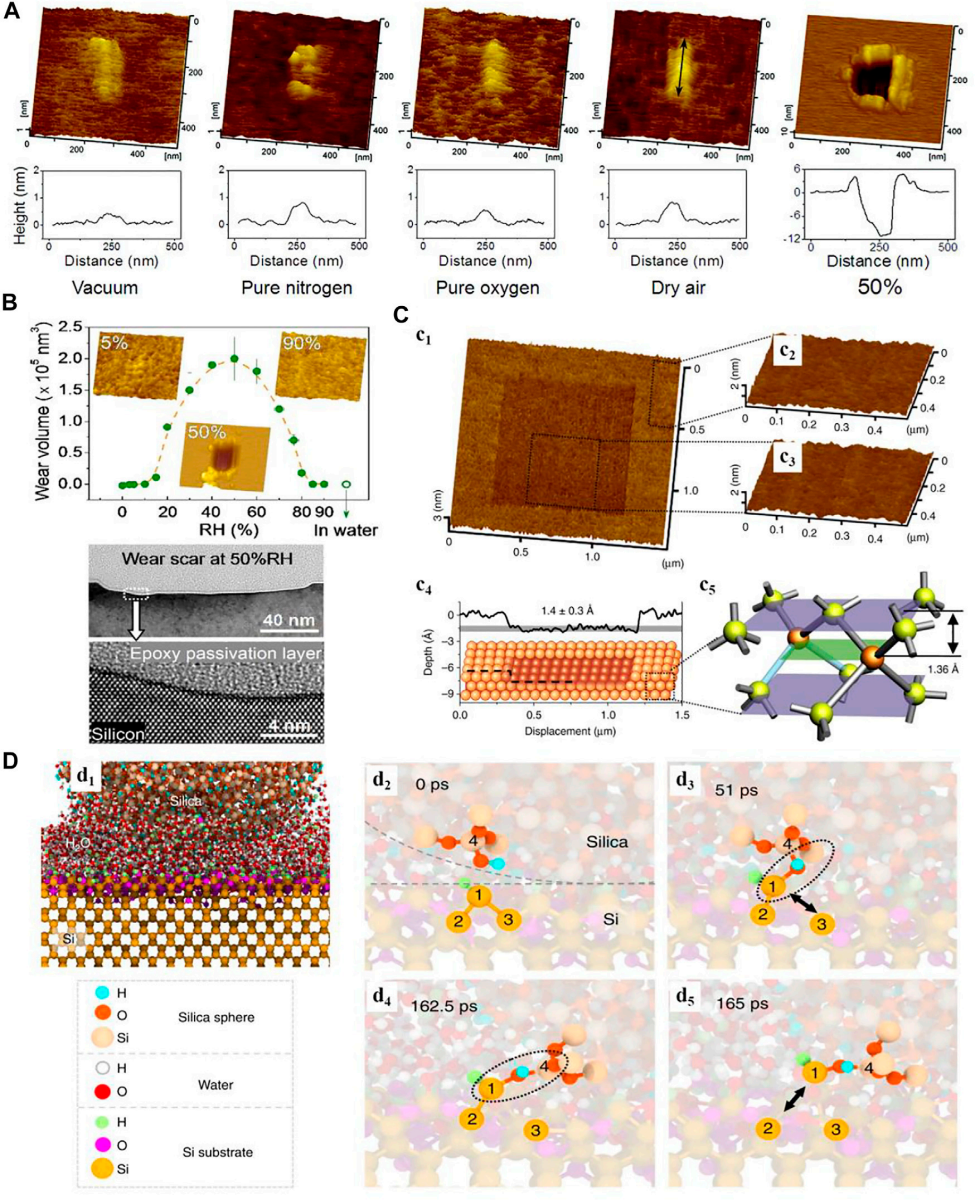
**(A)** Topographies of the wear scars on the silicon surface after sliding against silica microspheres in different environments. Adapted from Bingjun Yu et al., 2012 **(B)** RH-dependent tribochemical wear of silicon (upper) and TEM observations on the cross section of a tribochemical wear scar on the silicon surface. Adapted from Wang et al., 2015 **(C)** Single atomic layer removal of oxide-free Si(100) surface. Adapted from Lei Chen et al., 2018 **(D)** ReaxFF-MD simulation showing the processes of the interfacial bond forming and substrate bonds broken in a typical tribochemical wear of the Si surface against a silica nanosphere. Adapted from Lei Chen et al., 2018.

Because of their excellent mechanical and electronical properties, silicon and silicon oxide are widely used as structural and functional materials in the IC and MEMS after premanufacturing or processing (Achanta and Celis, 2007; Bhushan, 2007; Kim et al., 2007; Dong et al., 2014). Qian et al. have performed considerable studies and proved that the tribochemical reaction plays a dominant role in the nanowear of these materials especially when the tribological tests are operated in the conditions with water molecules (Jiaxin Yu et al., 2012; Cheng Chen et al., 2017). By taking the single crystalline silicon sliding silica microspheres as an example, we found that the surface wear behaviors in humid air instead of hillock form under dry (vacuum, pure nitrogen and oxygen, and dry air) conditions as the contact stress is below 2 GPa (Figure 3A) (Bingjun Yu et al., 2012). The contact stress is too low to induce silicon yield, so the material removal in humid is mainly due to the tribochemical reaction. The transmission electron microscopy (TEM) characterizations observed a perfect crystalline lattice even close to the worn surface, also supporting the occurrence of the tribochemical wear, rather than the mechanical wear (bottom images in Figure 3B). Wang et al. (2015) found that the tribochemical wear of silicon increases at first and then decreases as the RH ranges from 0% to 90% (upper images in Figure 3B). They proposed that the adsorption of solid-like water in low humid air (RH < 50%) is capable of facilitating the formation of interfacial Si–O–Si bonds, whereas the liquid-like water layer adsorbed under the high RH condition lubricates the sliding interface. These images have been proved by the MD simulations given by the Kubo group (Ootani et al., 2018), and the mechanism of interfacial bonding associated with the tribochemical removal has been widely applied in many of our experimental studies (Chen et al., 2015a; Chen et al., 2015b; Yan et al., 2019). In comparison, the tribochemical wear of the crystalline silicon occurs much more readily than that of silicon oxide due to the higher effective activation energy for the dissociation of Si–O bonds than Si–Si bonds (Lei Chen et al., 2017b). Zhaohui Liu et al. (2019) reported that the tribochemical wear of the silicon oxide surface increases but that of the crystalline silicon decreases with the increase in surrounding water temperature since the wettability of these two surfaces evolving at high temperature water alters the interfacial bonds forming.

Recently, we have achieved a region-specific removal of atomic layers on a single crystalline silicon surface *via* the tribochemical reactions (Figure 3C). The ReaxFF-MD simulations in Figure 3D demonstrate the silicon atom removal process. The silane groups at the two contact surfaces (d_2_) carry out the dehydration reaction following the formation of Si–O–Si bond across the sliding interface (d_3_), and then the Si–Si bond of the substrate is stretched and dissociated by the mechanical shear action (d_4_), finally leading to the removal of the Si atom from the substrate (d_5_) (Lei Chen et al., 2018). In addition, the ReaxFF-MD simulations, conducted by Ming Wang et al. (2019), Wang and Duan (2021), show that the formation of the interfacial Si–O–Si bonds at two amorphous silica surfaces may originate from the two different tribochemical reactions, one occurring between a silanol group and a surface Si–O–Si bond and the other occurring between the two silanol groups.

## Silicon-Based Ceramics and Glasses

**FIGURE 4:**
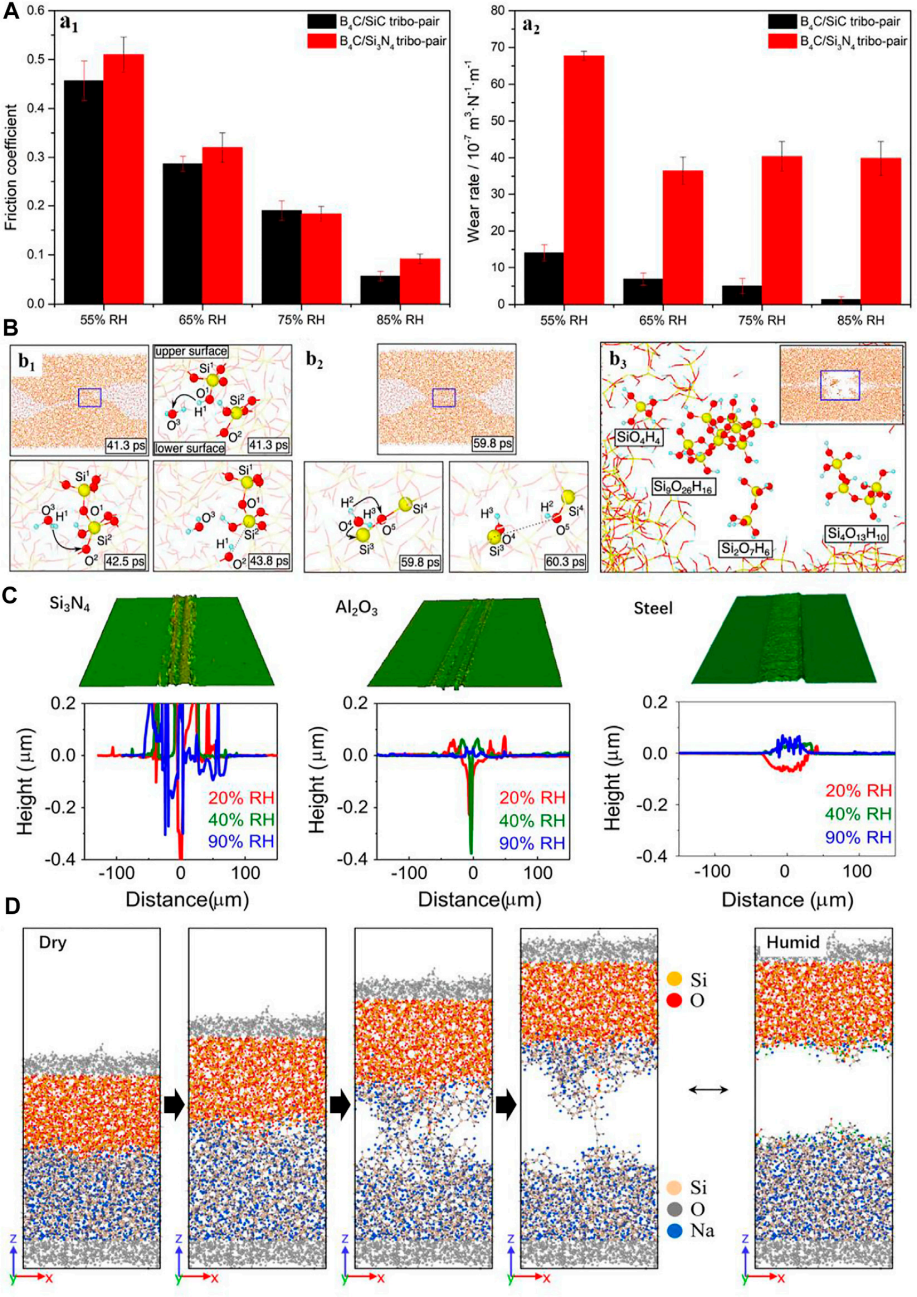
**(A)** Friction coefficients (a_1_) and wear rates (a_2_) of SiC and Si_3_N_4_ against B_4_C balls under various RH conditions. Adapted from Cao et al., 2020 **(B)** MD simulations of the self-mated sliding of SiC in water environment. (b_1_) Interfacial Si–O–Si bonds formed resulting in Si atom removal. (b_2_) Water molecule dissociating the interfacial Si–O–Si bonds. (b_3_) Formation of colloidal silica and hydrophilic hydrate particles. Adapted from Ootani et al., 2020b **(C)** Optical images and corresponding cross-section profiles of the wear tracks on the SLS glass surfaces against Si_3_N_4_, Al_2_O_3_, and stainless steel balls as the RH ranges from 20% to 90%. Adapted from He et al., 2015 **(D)** MD simulations showing the interfacial bonds between amorphous silica counter-surface and sodium silicate glass substrate formed under dry conditions and without the interfacial bond forming in water. Adapted from Hahn et al., 2020.

Silicon-based ceramics (such as Si_3_N_4_ and SiC) have been extensively used in the antiwear and lubrication systems due to the high hardness, potentially low friction, and excellent corrosion resistance (Dante and Kajdas, 2012; Sharma et al., 2016). However, the wear resistance of the silicon-based ceramics is challenged by the occurrence of the tribochemical reactions under complex operating environments in the real engineering applications (Renz et al., 2016; Das et al., 2018; Schmidt et al., 2019; Yue et al., 2019). It has been reported that the wear of Si_3_N_4_ and SiC ceramics against B_4_C balls can be suppressed in high RH conditions (Figure 4A) (Cao et al., 2020) due to the formation of a tribo-film in the tribochemical reactions occurring especially during the running-in period (Zum Gahr et al., 2001; Kovalčíková et al., 2014; Zhang et al., 2017). This mechanism that has been accepted broadly though the detailed structures of the tribo-film on the atomic scale is still unclear. Recently, Ootani et al., (2020a); Ootani et al., (2020b) have detected the tribochemical reaction process of self-mated sliding of SiC in water environment using the MD simulations. They clarified that Si–O–Si bonds were formed at the two contact surfaces; meanwhile, a double tribo-layer consisting of colloidal silica and hydrophilic hydrate particles was thus self-formed at the sliding interface (Figure 4B). Yang Wang et al. (2021) simulated the atomic-scale wear process of SiC against a SiO_2_ nano-sphere in a rolling contact state. They found that the wear of SiC is dominated by the interfacial adhesion–induced atom transfer from the original surface to the counterface in vacuum, whereas the adhesive wear of SiC was greatly reduced as the water molecules were added into the contact interface to form a third-body water layer which prevented the formation of interfacial bonds. In addition, the researchers have found that the self-mated SiC and Si_3_N_4_ show different tribological properties, which has a shorter running-in period for Si_3_N_4_ than that for SiC to enter the low-friction regime (Chen et al., 2001). Ootani et al. (2020a) further simulated the self-mated sliding of Si_3_N_4_ and SiC by using the first principles of MD. They revealed that similar formation of bridge Si–O–Si bonds was induced at the self-mated sliding interface of Si_3_N_4_ and SiC, but the tribochemical reaction is easily induced at the sliding interface of Si_3_N_4_ due to the easier dissociation for the Si–N bond than for the Si–C bond.

Apart from that, silicate glass is also one of the important silicon-based materials. An excellent surface quality of the optical components is a critical requirement, and the ultra-precision surface manufacturing is closely related to the tribochemical removal at the atomic level (Zhou et al., 2009; He et al., 2014; Yu et al., 2015; Jiang et al., 2017). Previous studies have found that the wear behavior of glass follows a stress corrosion theory. This model depicts that the molecules with proton donor sites and lone-pair orbitals (e.g., H_2_O) can enhance the dissociation of Si–O–Si network (crack growth) under tensile stress (Ciccotti, 2009; Bradley et al., 2013; Surdyka et al., 2014). Under a relatively low normal load, the tribochemical reaction dominated that material removal may occur on a glass surface. With the crystalline silicon, the tribochemical wear of the fused quartz glass against silica ball increases gradually as the RH increases (Bradley et al., 2013). Guo et al. (2020), Guo et al. (2021) further studied the wear process of the quartz glass at the atomic scale by MD simulations. They demonstrated that the surface atoms of the quartz glass are removed because of the synergistic action of the interfacial Si–O–Si bonding and mechanical shear action. Unexpectedly, we found that the wear depth of the soda-lime-silica (SLS) glass after sliding against the harder balls (Si_3_N_4_, Al_2_O_3_, and stainless steel) decreases with the increase of RH (Figure 4C) (He et al., 2015). Furthermore, we also found that the critical contact pressure for the wear process of the SLS glass is reduced because of the involving humidity (He et al., 2016a). He et al. (2016b) indicated that more water adsorption at higher RH can facilitate the formation of hydronium ion in the sodium-leached sites, which induced a local compressive stress and then enhanced the wear resistance of the SLS. Recently, Hahn et al. (2020) studied the role of H_2_O in the tribochemical reaction between the SiO_2_ sphere and SLS glass sliding interface using the ReaxFF-MD simulation. The results show that the primary role of H_2_O is to hydroxylate the silica and sodium silicate surface and suppress the formation of direct Si_silica_-O-Si_silicate_ interfacial bonds (Figure 4D). The formation of enormous hydroxyl groups in the interfacial region due to the dissociation of water molecules activated by sodium ions finally lead to an extremely weak wear.

## Metals

**FIGURE 5:**
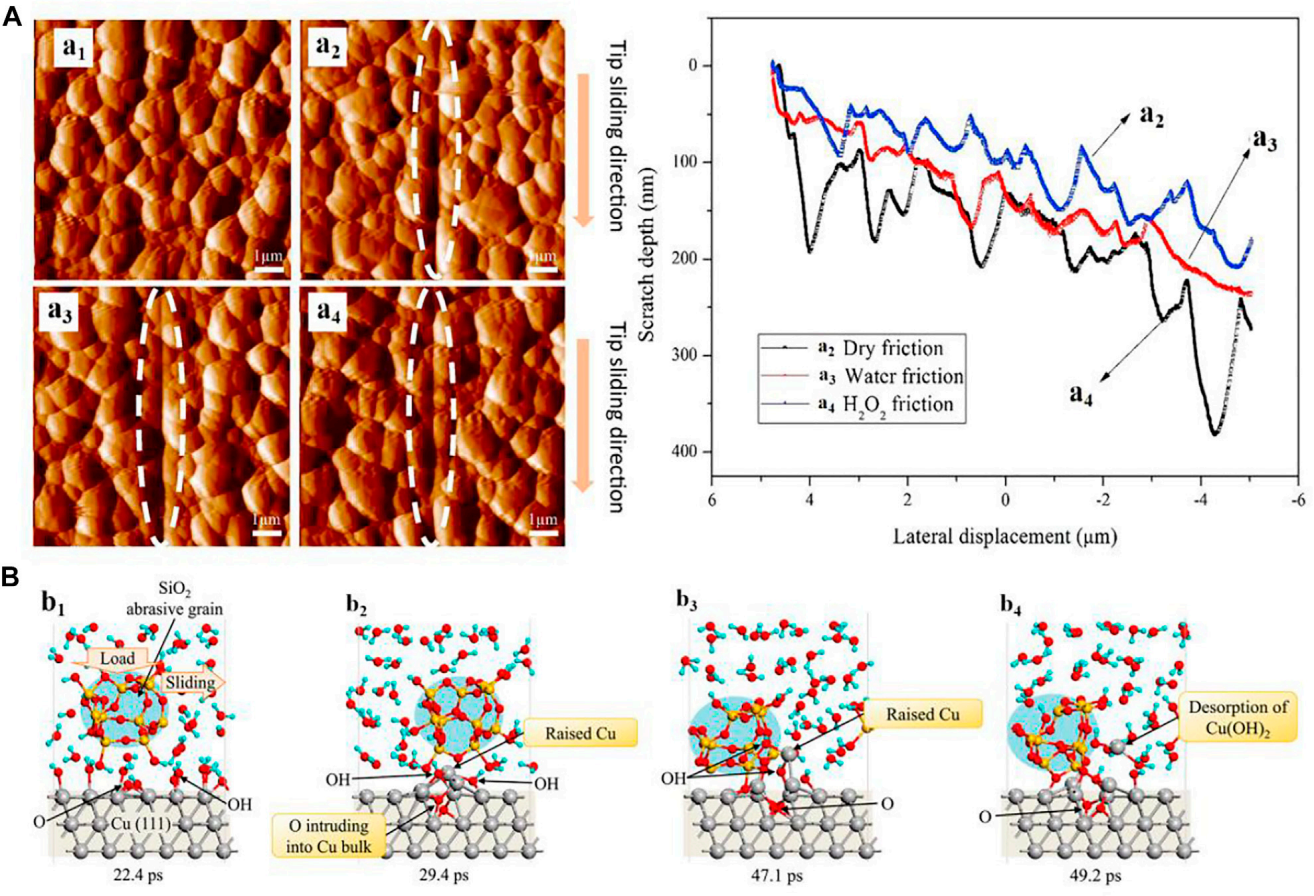
**(A)** Morphologies of the Al surfaces before (a_1_) and after the scratch tests in dry condition (a_2_), DI water (a_3_), and aqueous H_2_O_2_ (a_4_) with the profiles of scratch scars under the corresponding environments (right plots). Adapted from Yongguang Wang et al., 2019 **(B)** MD simulations showing the tribochemical wear process of Cu (111) sliding against a SiO_2_ abrasive grain in H_2_O_2_ solution. Adapted from Kawaguchi et al., 2016.

The material removal of metals or alloys at the micro/nanoscale has always been attracting considerable attention in ultra-precision manufacturing, such as chemical and mechanical polishing (CMP) (Chiu et al., 2003; Ahn et al., 2004; Zhang et al., 2016; Wang et al., 2017b; Guoqing Wang et al., 2021).Currently, the experimental studies using AFM (or nano-scratch tester) and MD simulations are normally carried out to detect the removal mechanism of metals against the single abrasive particle in the CMP process. For instance, Yongguang Wang et al. (2019) compared the nano-scratch wear of aluminum in dry, water, and H_2_O_2_ conditions and found that the surface wear of aluminum became more severe as the water or H_2_O_2_ molecules participated (Figure 5A). Similarly, Sharma et al. (2021) found that H_2_O_2_ in the CMP slurry can facilitate the nano-scratch wear of Cu compared to the conditions of humid air and deionized (DI) water. Kawaguchi et al. (2016) performed a tight-binding quantum chemical molecular dynamics (TBQC-MD) simulation to detect the atomic material removal process of Cu (111) surface sliding against a SiO_2_ abrasive grain in aqueous H_2_O_2_. As shown in Figure 5B, they demonstrated that H_2_O_2_ molecules react with Cu following the generation of hydroxide termination groups at the outermost surface (b_1_); then, O atoms intrude into the copper crystal cell internal to release Cu atoms by dissociating the Cu–Cu bonds (b_2_); and the interfacial bonds form between the released Cu atoms and the Si–OH surface terminations at SiO_2_ abrasive grain surface (b_3_); finally, the bonded Cu atoms are removed under the shear action following the formation of Cu(OH)_2_ products with further reaction with H_2_O_2_ (b_4_). Furthermore, the ReaxFF-MD simulations conducted by Guo et al. (2018)and Wen et al. (2019) showed that the Cu atoms are mainly removed in the form of clusters by the fracturing of Cu–Cu bonds and Cu–O bonds on the Cu substrate in the approximate CMP environments.

## Conclusion and Outlook

This article reviewed the recent advancements of the tribochemical wear mechanisms of typical materials in an ambient medium (such as gas, humidity, and water etc.) where the roles of the interfacial bonding are mainly considered in the material damage or removal. For the carbon materials (i.e., graphite and graphene, diamond, and DLC films), the interfacial C–C bonds is inhibited for achieving lower wear due to the passivation of the contact in gas or humidity. For the silicon-based materials (i.e., crystalline or amorphous silicon, silicon oxides, silicon-based ceramics, and glass), the wear behaviors are mainly determined by the capability of the interfacial bond bridges formed between the two solid contact surfaces, which are dependent on the surface chemistry of the counterface and surrounding atmosphere (such as humidity or water where water molecules must exist). The tribochemical wear of the silicon-based materials can be completely suppressed as the counterface is chemically inert or no water molecules participate. For some metals, such as Al and Cu, the atoms are more likely to be removed in the form of clusters by forming of interfacial bonds following the fracture of substrate bonds when the tribochemical reaction plays a dominant role. The involving medium could promote or inhibit the formation of the interfacial bonding and further change the micro-/nano-scale wear behaviors of these metals. Here, the abovementioned tribological issues indicate that the formation or fracture of the interfacial bonding bridges should play a critical role in the tribochemical wear behaviors of many frictional systems. Based on this, the atomic-scale wear (or materials removal) of the contact materials can be controlled by operating in an appropriate environmental or selecting proper medium, which is meaningful to avoid device failure, improve durability, and even develop the ultra-precision manufacture.

At present, the wear mechanism related to the interfacial bond forming is normally inferred based on the limited research studies. In recent years, an *in situ* transmission electron microscope (TEM) has been developed and applied to study the nanoscale and even the atomic wear through the direct analysis of the chemistry and bonding at the contacted interface. Nevertheless, the visual formation processes of the interfacial bonding is still impossible to verdict due to the technology restriction (Liao and Marks, 2017; Bernal and Carpick, 2019; Jacobs et al., 2019).In addition, most *in situ* TEM experiments, to date, have been performed in high vacuum so that the corresponding tests may not fully represent what takes place in an ambient medium. At the same time, more computation simulations for many engineering materials (such as MoS_2_, oxide ceramics, alloys, and so on) are needed to detect the wear mechanisms of these materials at the atomic scale. Moreover, the tribochemical wear depends not only on an ambient medium (humidity, water, and other liquid) but also on the surface chemical properties (substrate and counterface) and experimental factors (load, velocity, and temperature), so how to accurately predict the material wear and completely suppress the surface wear are still challenged.

## References

[B1] Achanta S. Celis J.-P. (2007). “Nanotribology of MEMS/NEMS,” in Fundamentals of Friction and Wear (Springer), 521–547. 10.1007/978-3-540-36807-6_23

[B2] Ahn Y. Yoon J.-Y. Baek C.-W. Kim Y.-K. (2004). Chemical Mechanical Polishing by Colloidal Silica-Based Slurry for Micro-scratch Reduction. Wear 257 (7-8), 785–789. 10.1016/j.wear.2004.03.020

[B3] Ayestarán Latorre C. Ewen J. P. Dini D. Righi M. C. (2021). Ab Initio insights into the Interaction Mechanisms between boron, Nitrogen and Oxygen Doped diamond Surfaces and Water Molecules. Carbon 171, 575–584. 10.1016/j.carbon.2020.09.044

[B4] Barthel A. J. Gregory M. D. Kim S. H. (2012). Humidity Effects on Friction and Wear between Dissimilar Metals. Tribol. Lett. 48 (3), 305–313. 10.1007/s11249-012-0026-5

[B5] Berman D. Erdemir A. Sumant A. V. (2014). Graphene: a New Emerging Lubricant. Mater. Today 17 (1), 31–42. 10.1016/j.mattod.2013.12.003

[B6] Berman D. Erdemir A. Sumant A. V. (2018). Approaches for Achieving Superlubricity in Two-Dimensional Materials. ACS nano 12 (3), 2122–2137. 10.1021/acsnano.7b09046 29522673

[B7] Bernal R. A. Carpick R. W. (2019). Visualization of Nanoscale Wear Mechanisms in Ultrananocrystalline diamond by Iin-Ssitu TEM Tribometry. Carbon 154, 132–139. 10.1016/j.carbon.2019.07.082

[B8] Bhushan B. (2007). Nanotribology and Nanomechanics of MEMS/NEMS and BioMEMS/BioNEMS Materials and Devices. Microelectronic Eng. 84 (3), 387–412. 10.1016/j.mee.2006.10.059

[B9] Bingjun Yu B. Li X. Dong H. Chen Y. Qian L. Zhou Z. (2012). Towards a Deeper Understanding of the Formation of Friction-Induced Hillocks on Monocrystalline Silicon. J. Phys. D: Appl. Phys. 45 (14), 145301. 10.1088/0022-3727/45/14/145301

[B10] Bradley L. C. Dilworth Z. R. Barnette A. L. Hsiao E. Barthel A. J. Pantano C. G. (2013). Hydronium Ions in Soda-Lime Silicate Glass Surfaces. J. Am. Ceram. Soc. 96 (2), 458–463. 10.1111/jace.12136

[B12] Cai Z. Zhu M. Shen H. Zhou Z. Jin X. (2009). Torsional Fretting Wear Behaviour of 7075 Aluminium alloy in Various Relative Humidity Environments. Wear 267 (1-4), 330–339. 10.1016/j.wear.2009.01.024

[B13] Cao C. Sun Y. Filleter T. (2014). Characterizing Mechanical Behavior of Atomically Thin Films: a Review. J. Mater. Res. 29 (3), 338–347. 10.1557/jmr.2013.339

[B14] Cao Y. Du L. Huang C. Liu W. Zhang W. (2011). Wear Behavior of Sintered Hexagonal boron Nitride under Atmosphere and Water Vapor Ambiences. Appl. Surf. Sci. 257 (23), 10195–10200. 10.1016/j.apsusc.2011.07.018

[B15] Cao X. Shang L. Liang Y. Lu Z. Zhang G. Xue Q. (2020). Tribological Performances of the boron Carbide Coatings Sliding against Silicon Carbide and Silicon Nitride Balls under Various Relative Humidity Conditions. Ceramics Int. 46 (3), 3074–3081. 10.1016/j.ceramint.2019.10.008

[B16] Chen M. Kato K. Adachi K. (2001). The Difference in Running-In Period and Friction Coefficient between Self-Mated Si_3_N_4_ and SiC under Water Lubrication. Tribol. Lett. 11 (1), 23–28. 10.1023/A:1016621929078

[B17] Chen L. He H. Wang X. Kim S. H. Qian L. (2015a). Tribology of Si/SiO2 in Humid Air: Transition from Severe Chemical Wear to Wearless Behavior at Nanoscale. Langmuir 31 (1), 149–156. 10.1021/la504333j 25521514

[B18] Chen L. Yang Y. J. He H. T. Kim S. H. Qian L. M. (2015b). Effect of Coadsorption of Water and Alcohol Vapor on the Nanowear of Silicon. Wear 332-333, 879–884. 10.1016/j.wear.2015.02.052

[B19] Chen W. Wang K. Liu X. He N. Xin H. Hao W. (2019). Investigation of the Friction and Wear Characteristics of Si3N4-hBN Ceramic Composites under marine Atmospheric Environment. Int. J. Refractory Met. Hard Mater. 81, 345–357. 10.1016/j.ijrmhm.2019.03.014

[B20] Cheng Chen C. Zhang P. Xiao C. Chen L. Qian L. (2017). Effect of Mechanical Interaction on the Tribochemical Wear of Bare Silicon in Water. Wear 376-377, 1307–1313. 10.1016/j.wear.2016.11.029

[B21] Chiu S.-Y. Wang Y.-L. Liu C.-P. Lan J.-K. Ay C. Feng M.-S. (2003). The Application of Electrochemical Metrologies for Investigating Chemical Mechanical Polishing of Al with a Ti Barrier Layer. Mater. Chem. Phys. 82 (2), 444–451. 10.1016/S0254-0584(03)00312-2

[B22] Ciccotti M. (2009). Stress-corrosion Mechanisms in Silicate Glasses. J. Phys. D: Appl. Phys. 42 (21), 214006. 10.1088/0022-3727/42/21/214006

[B23] Cui D.-D. Zhang L.-C. (2017). Nano-machining of Materials: Understanding the Process through Molecular Dynamics Simulation. Adv. Manuf. 5 (1), 20–34. 10.1007/s40436-016-0155-4

[B24] Curry J. F. Wilson M. A. Luftman H. S. Strandwitz N. C. Argibay N. Chandross M. (2017). Impact of Microstructure on MoS2 Oxidation and Friction. ACS Appl. Mater. Inter. 9 (33), 28019–28026. 10.1021/acsami.7b06917 28758391

[B25] Dante R. C. Kajdas C. K. (2012). A Review and a Fundamental Theory of Silicon Nitride Tribochemistry. Wear 288, 27–38. 10.1016/j.wear.2012.03.001

[B26] Das M. Bhimani K. Balla V. K. (2018). *In Vitro* tribological and Biocompatibility Evaluation of Sintered Silicon Nitride. Mater. Lett. 212, 130–133. 10.1016/j.matlet.2017.10.061

[B27] De Barros Bouchet M. I. Matta C. Vacher B. Le-Mogne T. Martin J. M. von Lautz J. (2015). Energy Filtering Transmission Electron Microscopy and Atomistic Simulations of Tribo-Induced Hybridization Change of Nanocrystalline diamond Coating. Carbon 87, 317–329. 10.1016/j.carbon.2015.02.041

[B28] De Fine M. Terrando S. Hintner M. Porporati A. A. Pignatti G. (2021). Pushing Ceramic-On-Ceramic in the Most Extreme Wear Conditions: a Hip Simulator Study. Orthopaedics Traumatol. Surg. Res. 107 (1), 102643. 10.1016/j.otsr.2020.05.003 32684432

[B29] Dimaki A. V. Shilko E. V. Dudkin I. V. Psakhie S. G. Popov V. L. (2020). Role of Adhesion Stress in Controlling Transition between Plastic, Grinding and Breakaway Regimes of Adhesive Wear. Sci. Rep. 10 (1), 1–13. 10.1038/s41598-020-57429-5 32005834 PMC6994689

[B30] Dong P. Chen Y.-K. Duan G.-H. Neilson D. T. (2014). Silicon Photonic Devices and Integrated Circuits. Nanophotonics 3 (4-5), 215–228. 10.1515/nanoph-2013-0023

[B31] Erdemir A. Martin J. M. (2018). Superior Wear Resistance of diamond and DLC Coatings. Curr. Opin. Solid State. Mater. Sci. 22 (6), 243–254. 10.1016/j.cossms.2018.11.003

[B32] Filho L. Schmidt S. Leifer K. Engqvist H. Högberg H. Persson C. (2019). Towards Functional Silicon Nitride Coatings for Joint Replacements. Coatings 9 (2), 73. 10.3390/coatings9020073

[B33] Fonseca D. J. Sequera M. (20112011). On MEMS Reliability and Failure Mechanisms. Int. J. Qual. Stat. Reliability 2011, 1–7. 10.1155/2011/820243

[B34] Fukuda K. Sheng S. L. Subhi Z. A. (2019). Tribological Behavior of Hydrophilic and Hydrophobic Surfaces in Atmosphere with Different Relative Humidity. Tribology Online 14 (5), 353–358. 10.2474/trol.14.353

[B35] Ge X. Li J. Zhang C. Liu Y. Luo J. (2019). Superlubricity and Antiwear Properties of In Situ-formed Ionic Liquids at Ceramic Interfaces Induced by Tribochemical Reactions. ACS Appl. Mater. Inter. 11 (6), 6568–6574. 10.1021/acsami.8b21059 30657308

[B36] Guo X. Wang X. Jin Z. Kang R. (2018). Atomistic Mechanisms of Cu CMP in Aqueous H2O2: Molecular Dynamics Simulations Using ReaxFF Reactive Force Field. Comput. Mater. Sci. 155, 476–482. 10.1016/j.commatsci.2018.09.022

[B37] Guo X. Huang J. Yuan S. Chen C. Jin Z. Kang R. (2020). Effect of Surface Hydroxylation on Ultra-precision Machining of Quartz Glass. Appl. Surf. Sci. 501, 144170. 10.1016/j.apsusc.2019.144170

[B38] Guo X. Huang J. Yuan S. Kang R. Guo D. (2021). Study Using ReaxFF-MD on the CMP Process of Fused Glass in Pure H2O/aqueous H2O2. Appl. Surf. Sci. 556, 149756. 10.1016/j.apsusc.2021.149756

[B39] Guoqing Wang G. Zhao G. Song J. Ding Q. (2021). Effect of Velocity and Interference Depth on the Tribological Properties of Alumina Sliding with Cu: a Molecular Dynamics Simulation. Chem. Phys. Lett. 775, 138669. 10.1016/j.cplett.2021.138669

[B40] Hahn S. H. Liu H. Kim S. H. Duin A. C. T. (2020). Atomistic Understanding of Surface Wear Process of Sodium Silicate Glass in Dry versus Humid Environments. J. Am. Ceram. Soc. 103 (5), 3060–3069. 10.1111/jace.17008

[B41] Hao Li H. Li X. Zhang G. Wang L. Wu G. (2017). Exploring the Tribophysics and Tribochemistry of MoS2 by Sliding MoS2/Ti Composite Coating under Different Humidity. Tribol. Lett. 65 (2), 38. 10.1007/s11249-017-0824-x

[B42] He H. Qian L. Pantano C. G. Kim S. H. (2014). Mechanochemical Wear of Soda Lime Silica Glass in Humid Environments. J. Am. Ceram. Soc. 97 (7), 2061–2068. 10.1111/jace.13014

[B43] He H. Qian L. Pantano C. G. Kim S. H. (2015). Effects of Humidity and Counter-surface on Tribochemical Wear of Soda-Lime-Silica Glass. Wear 342-343, 100–106. 10.1016/j.wear.2015.08.016

[B44] He H. Kim S. H. Qian L. (2016a). Effects of Contact Pressure, Counter-surface and Humidity on Wear of Soda-Lime-Silica Glass at Nanoscale. Tribology Int. 94, 675–681. 10.1016/j.triboint.2015.10.027

[B45] He H. Luo J. Qian L. Pantano C. G. Kim S. H. (2016b). Thermal Poling of Soda‐Lime Silica Glass with Nonblocking Electrodes-Part 2: Effects on Mechanical and Mechanochemical Properties. J. Am. Ceram. Soc. 99 (4), 1231–1238. 10.1111/jace.14080

[B46] He X. Meyer H. M. III Luo H. Qu J. (2021). Wear Penalty for Steel Rubbing against Hard Coatings in Reactive Lubricants Due to Tribochemical Interactions. Tribology Int. 160, 107010. 10.1016/j.triboint.2021.107010

[B48] Huang Y. Yao Q. Qi Y. Cheng Y. Wang H. Li Q. (2017). Wear Evolution of Monolayer Graphene at the Macroscale. Carbon 115, 600–607. 10.1016/j.carbon.2017.01.056

[B49] Huo L. Wang S. Pu J. Sun J. Lu Z. Ju P. (2018). Exploring the Low Friction of diamond-like Carbon Films in Carbon Dioxide Atmosphere by Experiments and First-Principles Calculations. Appl. Surf. Sci. 436, 893–899. 10.1016/j.apsusc.2017.12.044

[B50] Jacobs T. D. B. Greiner C. Wahl K. J. Carpick R. W. (2019). Insights into Tribology from *In Situ* Nanoscale Experiments. MRS Bull. 44 (6), 478–486. 10.1557/mrs.2019.122

[B51] Jiang C. Cheng J. Wu T. (2017). Theoretical Model of Brittle Material Removal Fraction Related to Surface Roughness and Subsurface Damage Depth of Optical Glass during Precision Grinding. Precision Eng. 49, 421–427. 10.1016/j.precisioneng.2017.04.004

[B52] Jiaxin Yu J. Kim S. H. Yu B. Qian L. Zhou Z. (2012). Role of Tribochemistry in Nanowear of Single-Crystalline Silicon. ACS Appl. Mater. Inter. 4 (3), 1585–1593. 10.1021/am201763z 22352895

[B53] Jingjing Wang J. Li X. Wu G. Lu Z. Zhang G. Xue Q. (2019). Origin of Low Friction for Amorphous Carbon Films with Different Hydrogen Content in Nitrogen Atmosphere. Tribology Int. 140, 105853. 10.1016/j.triboint.2019.105853

[B54] Kato K. Adachi K. (2002). Wear of Advanced Ceramics. Wear 253 (11), 1097–1104. 10.1016/S0043-1648(02)00240-5

[B55] Katsuki F. (2009). Single Asperity Tribochemical Wear of Silicon by Atomic Force Microscopy. J. Mater. Res. 24 (1), 173–178. 10.1557/Jmr.2009.0024

[B56] Kawaguchi K. Ito H. Kuwahara T. Higuchi Y. Ozawa N. Kubo M. (2016). Atomistic Mechanisms of Chemical Mechanical Polishing of a Cu Surface in Aqueous H2O2: Tight-Binding Quantum Chemical Molecular Dynamics Simulations. ACS Appl. Mater. Inter. 8 (18), 11830–11841. 10.1021/acsami.5b11910 27092706

[B57] Khanna R. Ong J. Oral E. Narayan R. (2017). Progress in Wear Resistant Materials for Total Hip Arthroplasty. Coatings 7 (7), 99. 10.3390/coatings7070099

[B58] Kim S. H. Asay D. B. Dugger M. T. (2007). Nanotribology and MEMS. Nano Today 2 (5), 22–29. 10.1016/S1748-0132(07)70140-8

[B59] Kim H.-J. Yoo S.-S. Kim D.-E. (2012). Nano-scale Wear: a Review. Int. J. Precis. Eng. Manuf. 13 (9), 1709–1718. 10.1007/s12541-012-0224-y

[B60] Kim H.-J. Seo K.-J. Kang K. H. Kim D.-E. (2016). Nano-lubrication: a Review. Int. J. Precis. Eng. Manuf. 17 (6), 829–841. 10.1007/s12541-016-0102-0

[B61] Kobatake S. Nakazawa S. Nagata K. Miyazawa S. Kawakubo Y. (2005). Pin-on-disk Wear Study on Thin-Film Disks for Contact Recording Systems. Microsyst. Technol. 11 (8), 921–924. 10.1007/s00542-005-0572-y

[B62] Kovalčíková A. Kurek P. Balko J. Dusza J. Šajgalík P. Mihaliková M. (2014). Effect of the Counterpart Material on Wear Characteristics of Silicon Carbide Ceramics. Int. J. Refractory Met. Hard Mater. 44, 12–18. 10.1016/j.ijrmhm.2014.01.006

[B63] Kumar N. Sharma N. Dash S. Popov C. Kulisch W. Reithmaier J. P. (2011). Tribological Properties of Ultrananocrystalline diamond Films in Various Test Atmosphere. Tribology Int. 44 (12), 2042–2049. 10.1016/j.triboint.2011.09.003

[B64] Kumar N. Ramadoss R. Kozakov A. T. Sankaran K. J. Dash S. Tyagi A. K. (2013). Humidity-dependent Friction Mechanism in an Ultrananocrystalline diamond Film. J. Phys. D: Appl. Phys. 46 (27), 275501. 10.1088/0022-3727/46/27/275501

[B65] Kumar K. M. Shanmuganathan P. V. Sethuramiah A. (2018). Tribology of Silicon Surfaces: a Review. Mater. Today Proc. 5 (11), 24809–24819. 10.1016/j.matpr.2018.10.279

[B66] Lee C. Li Q. Kalb W. Liu X.-Z. Berger H. Carpick R. W. (2010). Frictional Characteristics of Atomically Thin Sheets. science 328 (5974), 76–80. 10.1126/science.1184167 20360104

[B67] Lei Chen L. Qi Y. Yu B. Qian L. (2017a). Sliding Speed-dependent Tribochemical Wear of Oxide-free Silicon. Nanoscale Res. Lett. 12 (1), 404. 10.1186/s11671-017-2176-8 28610397 PMC5468177

[B68] Lei Chen L. Xiao C. He X. Yu B. Kim S. H. Qian L. (2017b). Friction and Tribochemical Wear Behaviors of Native Oxide Layer on Silicon at Nanoscale. Tribol. Lett. 65 (4), 1–8. 10.1007/s11249-017-0922-9

[B69] Lei Chen L. Wen J. Zhang P. Yu B. Chen C. Ma T. (2018). Nanomanufacturing of Silicon Surface with a Single Atomic Layer Precision via Mechanochemical Reactions. Nat. Commun. 9 (1), 1542. 10.1038/s41467-018-03930-5 29670215 PMC5906689

[B70] Levita G. Restuccia P. Righi M. C. (2016). Graphene and MoS2 Interacting with Water: A Comparison by Ab Initio Calculations. Carbon 107, 878–884. 10.1016/j.carbon.2016.06.072

[B71] Levita G. Righi M. C. (2017). Effects of Water Intercalation and Tribochemistry on MoS2 Lubricity: An Ab Initio Molecular Dynamics Investigation. ChemPhysChem 18 (11), 1475–1480. 10.1002/cphc.201601143 28067987

[B72] Li H. Zeng X. C. (2012). Wetting and Interfacial Properties of Water Nanodroplets in Contact with Graphene and Monolayer Boron-Nitride Sheets. ACS nano 6 (3), 2401–2409. 10.1021/nn204661d 22356158

[B73] Li Q. Zheng S. Pu J. Wang W. Li L. Wang L. (2019). Revealing the Failure Mechanism and Designing protection Approach for MoS2 in Humid Environment by First-Principles Investigation. Appl. Surf. Sci. 487, 1121–1130. 10.1016/j.apsusc.2019.05.215

[B74] Liao Y. Marks L. (2017). In Situsingle Asperity Wear at the Nanometre Scale. Int. Mater. Rev. 62 (2), 99–115. 10.1080/09506608.2016.1213942

[B75] Lin Wang L. Zhou P. Yan Y. Guo D. (2021). Investigation on Nanoscale Material Removal Process of BK7 and Fused Silica Glass during Chemical‐mechanical Polishing. Int. J. Appl. Glass. Sci. 12 (2), 198–207. 10.1111/ijag.15864

[B76] Lincong Liu L. Zhou M. Jin L. Li L. Mo Y. Su G. (2019). Recent Advances in Friction and Lubrication of Graphene and Other 2D Materials: Mechanisms and Applications. Friction 7 (3), 199–216. 10.1007/s40544-019-0268-4

[B77] Liu Y. Jiang Y. Sun J. Wang L. Liu Y. Chen L. (2021a). Durable Superlubricity of Hydrogenated diamond-like Carbon Film against Different Friction Pairs Depending on Their Interfacial Interaction. Appl. Surf. Sci. 560, 150023. 10.1016/j.apsusc.2021.150023

[B78] Liu Y. Chen L. Jiang B. Liu Y. Zhang B. Xiao C. (2021b). Origin of Low Friction in Hydrogenated diamond-like Carbon Films Due to Graphene Nanoscroll Formation Depending on Sliding Mode: Unidirection and Reciprocation. Carbon 173, 696–704. 10.1016/j.carbon.2020.11.039

[B79] Manimunda P. Al-Azizi A. Kim S. H. Chromik R. R. (2017). Shear-induced Structural Changes and Origin of Ultralow Friction of Hydrogenated diamond-like Carbon (DLC) in Dry Environment. ACS Appl. Mater. Inter. 9 (19), 16704–16714. 10.1021/acsami.7b03360 28459534

[B80] Marchetto D. Held C. Hausen F. Wählisch F. Dienwiebel M. Bennewitz R. (2012). Friction and Wear on Single-Layer Epitaxial Graphene in Multi-Asperity Contacts. Tribol. Lett. 48 (1), 77–82. 10.1007/s11249-012-9945-4

[B81] Martini A. Eder S. J. Dörr N. (2020). Tribochemistry: a Review of Reactive Molecular Dynamics Simulations. Lubricants 8 (4), 44. 10.3390/lubricants8040044

[B82] Ming Wang M. Duan F. Mu X. (2019). Effect of Surface Silanol Groups on Friction and Wear between Amorphous Silica Surfaces. Langmuir 35 (16), 5463–5470. 10.1021/acs.langmuir.8b04291 30925219

[B83] Ootani Y. Xu J. Hatano T. Kubo M. (2018). Contrasting Roles of Water at Sliding Interfaces between Silicon-Based Materials: First-Principles Molecular Dynamics Sliding Simulations. J. Phys. Chem. C 122 (19), 10459–10467. 10.1021/acs.jpcc.8b01953

[B84] Ootani Y. Xu J. Adachi K. Kubo M. (2020a). First-principles Molecular Dynamics Study of Silicon-Based Ceramics: Different Tribochemical Reaction Mechanisms during the Running-In Period of Silicon Nitride and Silicon Carbide. J. Phys. Chem. C 124 (37), 20079–20089. 10.1021/acs.jpcc.0c04613

[B85] Ootani Y. Xu J. Takahashi N. Akagami K. Sakaki S. Wang Y. (2020b). Self-formed Double Tribolayers Play Collaborative Roles in Achieving Superlow Friction in an Aqueous Environment. J. Phys. Chem. C 124 (15), 8295–8303. 10.1021/acs.jpcc.0c02068

[B86] Picas J. A. Forn A. Baile M. Martín E. (2006). Humidity Effect on Friction and Wear Behaviour of Self-Lubricant Coatings. Surf. Eng. 22 (4), 314–319. 10.1179/174329406X98458

[B87] Popov V. L. Psakhie S. G. (2007). Numerical Simulation Methods in Tribology. Tribology Int. 40 (6), 916–923. 10.1016/j.triboint.2006.02.020

[B88] Psakhie S. Shilko E. Smolin A. Astafurov S. Ovcharenko V. (2013). Development of a Formalism of Movable Cellular Automaton Method for Numerical Modeling of Fracture of Heterogeneous Elastic-Plastic Materials. Frattura Ed. Integrita Strutturale 7 (24), 26–59. 10.3221/igf-esis.24.04

[B89] Qi Y. Liu J. Zhang J. Dong Y. Li Q. (2017). Wear Resistance Limited by Step Edge Failure: the Rise and Fall of Graphene as an Atomically Thin Lubricating Material. ACS Appl. Mater. Inter. 9 (1), 1099–1106. 10.1021/acsami.6b12916 28073278

[B90] Qi Y. Liu J. Dong Y. Feng X.-Q. Li Q. (2018). Impacts of Environments on Nanoscale Wear Behavior of Graphene: Edge Passivation vs. Substrate Pinning. Carbon 139, 59–66. 10.1016/j.carbon.2018.06.029

[B91] Qin W. Yue W. Wang C. (2018). Controllable Wear Behaviors of Silicon Nitride Sliding against Sintered Polycrystalline diamond via Altering Humidity. J. Am. Ceram. Soc. 101 (6), 2506–2515. 10.1111/jace.15421

[B92] Rajak D. K. Kumar A. Behera A. Menezes P. L. (2021). Diamond-like Carbon (DLC) Coatings: Classification, Properties, and Applications. Appl. Sci. 11 (10), 4445. 10.3390/app11104445

[B93] Rani R. Panda K. Kumar N. Sankaran K. J. Pandian R. Ficek M. (2018a). Triboenvironment Dependent Chemical Modification of Sliding Interfaces in Ultrananocrystalline Diamond Nanowall Film: Correlation with Friction and Wear. J. Phys. Chem. C 122 (1), 945–956. 10.1021/acs.jpcc.7b10992

[B94] Rani R. Panda K. Kumar N. Titovich K. A. Ivanovich K. V. Vyacheslavovich S. A. (2018b). Tribological Properties of Ultrananocrystalline diamond Films: Mechanochemical Transformation of Sliding Interfaces. Sci. Rep. 8 (1), 1–16. 10.1038/s41598-017-18425-4 29321546 PMC5762651

[B95] Renz A. Khader I. Kailer A. (2016). Tribochemical Wear of Cutting-Tool Ceramics in Sliding Contact against a Nickel-Base alloy. J. Eur. Ceram. Soc. 36 (3), 705–717. 10.1016/j.jeurceramsoc.2015.10.032

[B96] Rietsch J.-C. Brender P. Dentzer J. Gadiou R. Vidal L. Vix-Guterl C. (2013). Evidence of Water Chemisorption during Graphite Friction under Moist Conditions. Carbon 55, 90–97. 10.1016/j.carbon.2012.12.013

[B97] Seo J. (2021). A Review on Chemical and Mechanical Phenomena at the Wafer Interface during Chemical Mechanical Planarization. J. Mater. Res. 36 (1), 235–257. 10.1557/s43578-020-00060-x

[B98] Sharma S. K. Kumar B. V. M. Kim Y.-W. (2016). Tribological Behavior of Silicon Carbide Ceramics - A Review. J. Korean Ceram. Soc. 53 (6), 581–596. 10.4191/kcers.2016.53.6.581

[B99] Sharma M. Chen C.-C. A. Gupta A. (2021). Material Removal and Wear Behaviour of Copper Thin Film in Ambient Air and Wet Environment by Nanoindenter. ECS J. Solid State. Sci. Technol. 10 (5), 054001. 10.1149/2162-8777/abfb0d

[B100] Shi J. Gong Z. Wang C. Zhang B. Zhang J. (2017a). Tribological Properties of Hydrogenated Amorphous Carbon Films in Different Atmospheres. Diamond Relat. Mater. 77, 84–91. 10.1016/j.diamond.2017.06.005

[B101] Shi J. Gong Z. Wang Y. Gao K. Zhang J. (2017b). Friction and Wear of Hydrogenated and Hydrogen-free diamond-like Carbon Films: Relative Humidity Dependent Character. Appl. Surf. Sci. 422, 147–154. 10.1016/j.apsusc.2017.05.210

[B102] Spear J. C. Ewers B. W. Batteas J. D. (2015). 2D-nanomaterials for Controlling Friction and Wear at Interfaces. Nano Today 10 (3), 301–314. 10.1016/j.nantod.2015.04.003

[B103] Surdyka N. D. Pantano C. G. Kim S. H. (2014). Environmental Effects on Initiation and Propagation of Surface Defects on Silicate Glasses: Scratch and Fracture Toughness Study. Appl. Phys. A. 116 (2), 519–528. 10.1007/s00339-014-8552-7

[B104] Tyagi A. Walia R. S. Murtaza Q. Pandey S. M. Tyagi P. K. Bajaj B. (2019). A Critical Review of diamond like Carbon Coating for Wear Resistance Applications. Int. J. Refractory Met. Hard Mater. 78, 107–122. 10.1016/j.ijrmhm.2018.09.006

[B11] Uzoma P. C. Hu H. Khadem M. Oleksiy V. P. (2020). Tribology of 2D Nanomaterials: A Review[J]. Coatings 10 (9), 897. 10.3390/coatings10090897

[B105] Vahdat V. Ryan K. E. Keating P. L. Jiang Y. Adiga S. P. Schall J. D. (2014). Atomic-scale Wear of Amorphous Hydrogenated Carbon during Intermittent Contact: a Combined Study Using experiment, Simulation, and Theory. ACS nano 8 (7), 7027–7040. 10.1021/nn501896e 24922087

[B106] Wang L. Duan F. (2018). Nanoscale Wear Mechanisms of Few-Layer Graphene Sheets Induced by Interfacial Adhesion. Tribology Int. 123, 266–272. 10.1016/j.triboint.2018.02.045

[B107] Wang M. Duan F. (2021). Atomic-Level Material Removal Mechanisms of Si(110) Chemical Mechanical Polishing: Insights from ReaxFF Reactive Molecular Dynamics Simulations. Langmuir 37 (6), 2161–2169. 10.1021/acs.langmuir.0c03416 33530684

[B108] Wang S. Komvopoulos K. (2021). A Molecular Dynamics Study of the Oxidation Mechanism, Nanostructure Evolution, and Friction Characteristics of Ultrathin Amorphous Carbon Films in Vacuum and Oxygen Atmosphere. Sci. Rep. 11 (1), 3914. 10.1038/s41598-021-81659-w 33594088 PMC7886871

[B109] Wang X. D. Song C. F. Yu B. J. Chen L. Qian L. M. (2013). Nanowear Behaviour of Monocrystalline Silicon against SiO2 Tip in Water. Wear 298-299, 80–86. 10.1016/j.wear.2012.12.049

[B110] Wang X. Kim S. H. Chen C. Chen L. He H. Qian L. (2015). Humidity Dependence of Tribochemical Wear of Monocrystalline Silicon. ACS Appl. Mater. Inter. 7 (27), 14785–14792. 10.1021/acsami.5b03043 26098989

[B47] Wang X. Wang P. Zhang B. Yang S. Zhang J. (2009). The Tribological Properties of Fullerene-like Hydrogenated Carbon (FL-C: H) Film under Different Humidity Conditions. Tribol. T. 52 (3), 354–359. 10.1080/10402000802563125

[B111] Wang Y. Xu J. Ootani Y. Bai S. Higuchi Y. Ozawa N. (2017a). Tight-binding Quantum Chemical Molecular Dynamics Study on the Friction and Wear Processes of diamond-like Carbon Coatings: Effect of Tensile Stress. ACS Appl. Mater. Inter. 9 (39), 34396–34404. 10.1021/acsami.7b07551 28914057

[B112] Wang Y. Chen Y. Zhao Y. Min P. Qi F. Liu X. (2017b). Chemical Mechanical Planarization of Al alloy in Alkaline Slurry at Low Down Pressure. J. Mater. Sci. Mater. Electron. 28 (4), 3364–3372. 10.1007/s10854-016-5930-2

[B113] Wang Y. Su Y. Zhang J. Chen Q. Xu J. Bai S. (2020). Reactive Molecular Dynamics Simulations of Wear and Tribochemical Reactions of Diamond like Carbon Interfaces with Nanoscale Asperities under H2 Gas: Implications for Solid Lubricant Coatings. ACS Appl. Nano Mater. 3 (7), 7297–7304. 10.1021/acsanm.0c01775

[B114] Wei B. Kong N. Zhang J. Li H. Hong Z. Zhu H. (2021). A Molecular Dynamics Study on the Tribological Behavior of Molybdenum Disulfide with Grain Boundary Defects during Scratching Processes. Friction 9 (5), 1198–1212. 10.1007/s40544-020-0459-z

[B115] Wen J. Ma T. Zhang W. van Duin A. C. T. van Duin D. M. Hu Y. (2019). Atomistic Insights into Cu Chemical Mechanical Polishing Mechanism in Aqueous Hydrogen Peroxide and glycine: ReaxFF Reactive Molecular Dynamics Simulations. J. Phys. Chem. C 123 (43), 26467–26474. 10.1021/acs.jpcc.9b08466

[B116] Windom B. C. Sawyer W. G. Hahn D. W. (2011). A Raman Spectroscopic Study of MoS2 and MoO3: Applications to Tribological Systems. Tribol. Lett. 42 (3), 301–310. 10.1007/s11249-011-9774-x

[B117] Wu D. Ren S. Pu J. Lu Z. Zhang G. Wang L. (2018). A Comparative Study of Tribological Characteristics of Hydrogenated DLC Film Sliding against Ceramic Mating Materials for Helium Applications. Appl. Surf. Sci. 441, 884–894. 10.1016/j.apsusc.2018.01.206

[B118] Xinchun Chen X. Zhang C. Kato T. Yang X.-a. Wu S. Wang R. (2017). Evolution of Tribo-Induced Interfacial Nanostructures Governing Superlubricity in A-C:H and a-C:H:Si Films. Nat. Commun. 8 (1), 1–13. 10.1038/s41467-017-01717-8 29162811 PMC5698435

[B119] Yan W. Gong J. Yu B. Chen L. Qian L. (2019). Self-lubrication of Si/SiO2 Interface Achieved through Running-In at Low Sliding Speed. Wear 426-427, 828–834. 10.1016/j.wear.2019.01.117

[B120] Yang Wang Y. Yukinori K. Koike R. Ootani Y. Adachi K. Kubo M. (2021). Selective Wear Behaviors of a Water-Lubricating SiC Surface under Rotating-Contact Conditions Revealed by Large-Scale Reactive Molecular Dynamics Simulations. J. Phys. Chem. C 125 (27), 14957–14964. 10.1021/acs.jpcc.1c02765

[B121] Yongguang Wang Y. Zhu Y. Zhao D. Bian D. (2019). Nanoscratch of Aluminum in Dry, Water and Aqueous H2O2 Conditions. Appl. Surf. Sci. 464, 229–235. 10.1016/j.apsusc.2018.09.075

[B122] Yu J. Yuan W. Hu H. Zang H. Cai Y. Ji F. (2015). Nanoscale Friction and Wear of Phosphate Laser Glass and BK7 Glass against Single CeO2 Particle by AFM. J. Am. Ceram. Soc. 98 (4), 1111–1120. 10.1111/jace.13356

[B123] Yu Q. Chen X. Zhang C. Luo J. (2020). Influence Factors on Mechanisms of Superlubricity in DLC Films: A Review. Front. Mech. Eng. 6, 65. 10.3389/fmech.2020.00065

[B124] Yue T. Yue W. Qin W. Liu P. Wang C. (2019). Effects of Environmental Atmospheres on Tribological Behaviors of Sintered Polycrystalline diamond Sliding against Silicon Nitride. Int. J. Refractory Met. Hard Mater. 81, 85–93. 10.1016/j.ijrmhm.2019.02.023

[B125] Yunhai Liu Y. Chen L. Zhang B. Cao Z. Shi P. Peng Y. (2019). Key Role of Transfer Layer in Load Dependence of Friction on Hydrogenated diamond-like Carbon Films in Humid Air and Vacuum. Materials 12 (9), 1550. 10.3390/ma12091550 PMC6539659 31083600

[B126] Zhang J. Liu Y. Yan C. Zhang W. (2016). Investigation on Chemical Mechanical Planarization Performance of the Replacement Metal Gate Aluminum Polishing Slurry. ECS J. Solid State. Sci. Technol. 5 (7), P446–P450. 10.1149/2.0291607jss

[B127] Zhang D. Chen W. Ai X. Lv Z.-l. (2017). Effect of the Humidity on the Friction and Wear Characteristics of Si3N4-hBN Composite Ceramics. Proc. Inst. Mech. Eng. J: J. Eng. Tribology 231 (12), 1517–1526. 10.1177/1350650117700342

[B128] Zhang J. Chen X. Xu Q. Ma T. Hu Y. Wang H. (2019). Effects of Grain Boundary on Wear of Graphene at the Nanoscale: A Molecular Dynamics Study. Carbon 143, 578–586. 10.1016/j.carbon.2018.11.067

[B129] Zhang L. C. Mylvaganam K. (2006). Nano-Tribological Analysis by Molecular Dynamics Simulation-A Review. J Comput. Theor. Nanosci 3 (2), 167–188. 10.1166/jctn.2006.2999

[B130] Zhaohui Liu Z. Gong J. Xiao C. Shi P. Kim S. H. Chen L. (2019). Temperature-Dependent Mechanochemical Wear of Silicon in Water: The Role of Si-OH Surfacial Groups. Langmuir 35 (24), 7735–7743. 10.1021/acs.langmuir.9b00790 31126172

[B131] Zhe Chen Z. He X. Xiao C. Kim S. (2018). Effect of Humidity on Friction and Wear-A Critical Review. Lubricants 6 (3), 74. 10.3390/lubricants6030074

[B132] Zheng F. Duan F. (2019). Atomistic Mechanism of the Weakened Wear Resistance of Few-Layer Graphene Induced by point Defects. Tribology Int. 134, 87–92. 10.1016/j.triboint.2019.01.035

[B133] Zheng-yang Li Z.-y. Yang W.-j. Wu Y.-p. Wu S.-b. Cai Z.-b. (2017). Role of Humidity in Reducing the Friction of Graphene Layers on Textured Surfaces. Appl. Surf. Sci. 403, 362–370. 10.1016/j.apsusc.2017.01.226

[B134] Zhou L. Shiina T. Qiu Z. Shimizu J. Yamamoto T. Tashiro T. (2009). Research on Chemo-Mechanical Grinding of Large Size Quartz Glass Substrate. Precision Eng. 33 (4), 499–504. 10.1016/j.precisioneng.2009.01.006

[B135] Zum Gahr K.-H. Blattner R. Hwang D.-H. Pöhlmann K. (2001). Micro-and Macro-Tribological Properties of SiC Ceramics in Sliding Contact. Wear 250 (1-12), 299–310. 10.1016/S0043-1648(01)00595-6

